# The perspectives of UK personnel towards current killing practices for laboratory rodents

**DOI:** 10.1038/s41598-023-31808-0

**Published:** 2023-03-23

**Authors:** Jasmine M. Clarkson, Matthew C. Leach, Dorothy E. F. McKeegan, Jessica E. Martin

**Affiliations:** 1 Institute of Biodiversity, One Health and Veterinary Medicine, College of Medical Veterinary and Life Sciences, University of Glasgow, Glasgow, Scotland; 2 School of Natural and Environmental Sciences, Newcastle University, Newcastle Upon Tyne, England; 3 Present Address: Comparative Biology Centre, School of Natural and Environmental Sciences, Newcastle University, Newcastle Upon Tyne, England; 4 The Royal (Dick) School of Veterinary Studies and The Roslin Institute, The University of Edinburgh, Edinburgh, Scotland

**Keywords:** Experimental models of disease, Animal disease models, Human behaviour

## Abstract

Rodents are the predominant species used for scientific research and must be humanely killed upon completion of the work. In the UK this is regulated by Schedule 1 of the Animals Scientific Procedures Act 1986, which lists permitted methodologies considered capable of humane killing, including overdose of an anaesthetic, exposure to carbon dioxide (CO_2_) gas, dislocation of the neck and concussion of the brain by striking the cranium. Although all are permitted, operator motivations behind method selection and individual operator preference remain unknown. The views of 219 laboratory animal personnel on institutional availability and use of Schedule 1 killing methods for laboratory rodents were obtained. Only 10% of participants reported that all four methods were available at their institution with 57.5% of respondents preferring cervical dislocation. For CO_2_, only 18.6% of participants reported using the recommended flow rate, while 45.5% did not know the flow rate employed. We highlight the urgent requirement for the development of quality-controlled training programmes, to improve knowledge and confidence in the selection and application of killing methods. We advocate for continuous review of killing practices to ensure best practice is reflected in legislation and achieve optimal protection of the welfare of laboratory rodents during killing.

## Introduction

Millions of rodents are used for biomedical purposes each year^1^ and almost all must be killed in line with existing national legislation upon completion of the scientific work. In the United Kingdom, killing laboratory animals is regulated by Animals (Scientific) Procedures Act 1986 (ASPA) section 15A and Schedule 1, which lists appropriate methods considered to constitute humane means of killing^2^. For laboratory rodents these include overdose of an anaesthetic using an appropriate agent and route, exposure to carbon dioxide (CO_2_) gas in a rising concentration, dislocation of the neck (for rodents up to 500 g) and concussion of the brain by striking the cranium (for rodents up to 1 kg; also referred to as blunt force trauma). The intentional act of killing an animal has several ethical implications and can impact both animal and operator welfare. As such, the choice of killing method is multifaceted, encompassing its welfare impact on the animal and effects on potential scientific outcomes (either due to their chemical properties (e.g., anaesthetics and CO_2_) or associated tissue damage (e.g., cervical dislocation and concussion))^3^. The choice of killing method may also be influenced by a desire to minimise the emotional impact on personnel responsible for killing the animals, promoting workflow efficiency and convenience, availability of competency and training programmes and health and safety considerations^3^. To our knowledge, there are no published studies exploring the motivations behind killing method selection in rodents and there are also no published guidelines aiding method selection in a range of scenarios.

Adherence to the principles of the 3Rs is a fundamental component underpinning the use of animals for scientific purposes, aiming to minimise unnecessary suffering and protect animal welfare. Such values are also vital for public acceptance of animal research and support for continued funding. Although the exact definition of ‘humane’ is deliberated, generally it is considered to mean minimising the harm inflicted and/or avoiding unnecessary pain, suffering or distress^12,13^. A significant and growing body of evidence has demonstrated that exposure to CO_2_ is aversive to laboratory rodents^4–8^. This includes studies demonstrating increased stress and anxiety, negative sensations such as dyspnoea (breathlessness) and even pain at high concentrations (> 40%)^9–11^. As such, legitimate questions may be posed surrounding its continued inclusion in legislation (Schedule 1 ASPA)^2^, but whether and how these concerns have impacted the preferences and application of this methodology across laboratory personnel has not been studied. One argument for its continued use is that currently there is a lack of a more humane high throughput alternative available. Further, as highlighted in a recent review^14^ the remaining permitted methodologies such as overdose of anaesthetic, cervical dislocation and blunt force trauma are understudied with regard to their ability to provide a humane death and therefore aren’t without animal welfare concern due to their potential for inaccuracy and high failure rates^14,15^. Notwithstanding growing concern surrounding whether all methods listed in Schedule 1 are actually capable of inducing a humane death^4,5^, a lack of consistency in method application and choice across institutions could also result in unnecessary suffering during killing. The wider perception of the use of animals for scientific purposes, especially around killing, is an essential component of public trust in science, and is threatened by continued use of any killing method considered aversive or associated with poor welfare outcomes^16,17^.

The purpose of this survey was to characterise, for the first time, the current situation in UK research establishments regarding the availability all Schedule 1 killing methods for rodents, and in the case of CO_2_, the equipment and methods used. We aimed to determine the attitudes of those engaged in this work towards all of the permitted methods included in Schedule 1 legislation and examine the effects of selected demographic factors (e.g., job role, sector, experience) on their preferences. Our overall aim was to shed light on this crucial element of research animal use, to inform further refinement of killing methods used for laboratory rodents and staff training, both of which contribute to improved welfare at killing.

## Results

### Participant demographics

**Figure 1:**
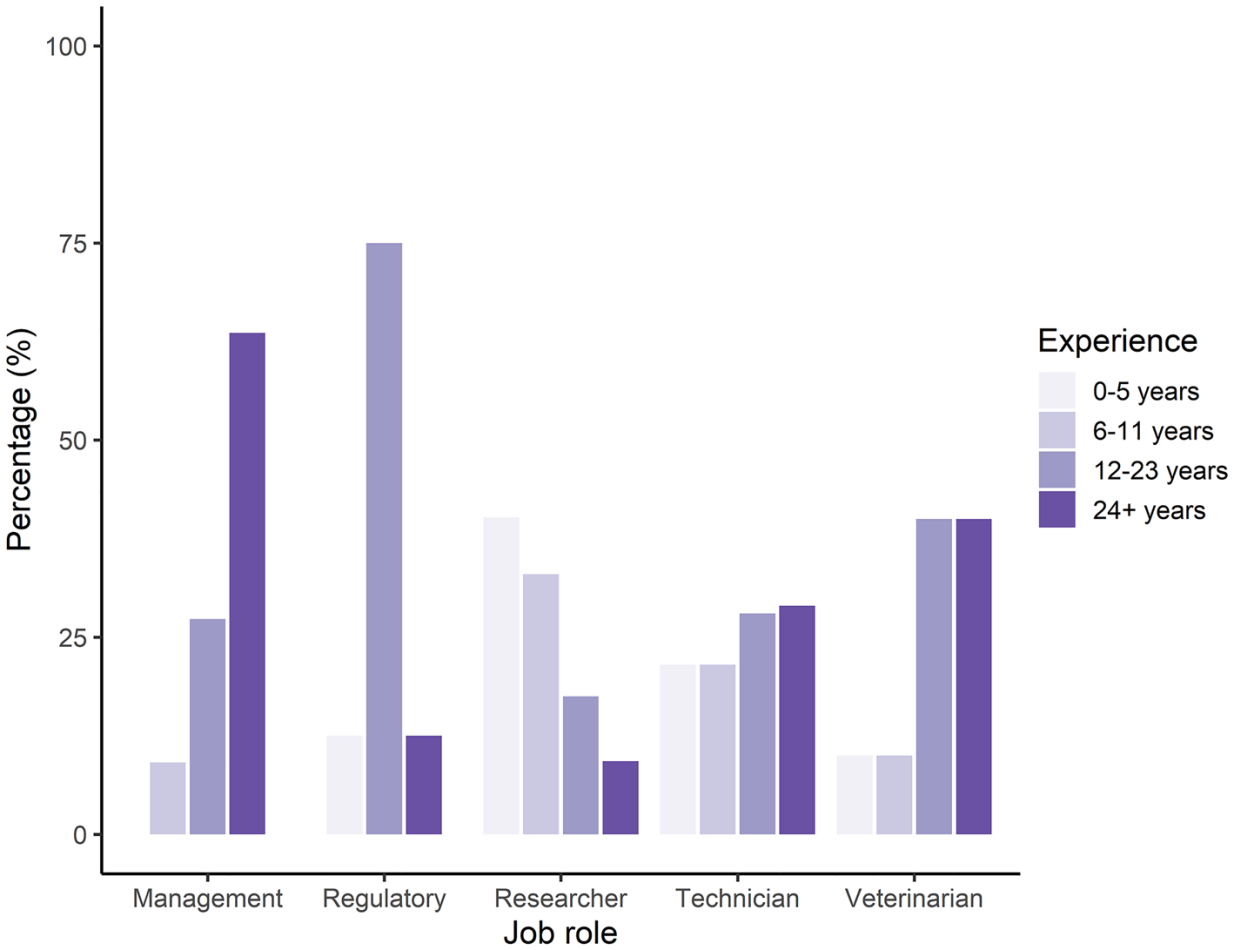
Percentage (%) of a total of 219 participant responses within each current job role according to their duration of experience of working with laboratory rodents.

A total of 219 participants completed the survey, with most responses coming from the academic sector (87.2%; n = 191/219) with a smaller proportion of responses coming from contract research organisations (7.8%, n = 17/219), pharmaceutical (2.7%, n = 6/219) and other (2.3%, n = 5/219) sectors. Overall, in terms of current role, 44.3% (n = 97/219) of responses came from researchers, 42.5% (n = 93) from technicians, 5% (n = 11/219) from management, 4.6% (n = 10/219) from veterinarians and 3.7% (n = 8/219) from participants with a regulatory role (e.g., Named Training and Competency Officer or unit managers). We also found a wide and relatively balanced range in experience level across participants (0–5 years 27.9% (n = 61); 6–11 years 24.7% (n = 54/219); 12–23 years 25.6% (n = 56/219); and 24 + years 21.9% (n = 48/219). Figure 1 highlights the spread of experience levels within each job role, showing that technicians had the most balanced range in experience. Researchers reported lower experience levels, whereas individuals in management, regulatory and veterinary roles reported higher experience levels. Most participants predominantly work with mice (86.8%, n = 190/219); followed by rats (11%, n = 24), hamsters (0.5%, n = 1/219) or gerbils (0.5%, n = 1/219). We also asked how often, on average, participants kill laboratory rodents, with 11.4% (n = 25/219) reporting this was daily, 19.6% (n = 43/219) once a week, 29.7% (n = 65/219) a couple of times a week, 23.7% (n = 52/219) monthly, 9.1% (n = 20/219) a couple of times a year and 6.4% (n = 14/219) almost never.

### Schedule 1 killing method availability and use

**Figure 2:**
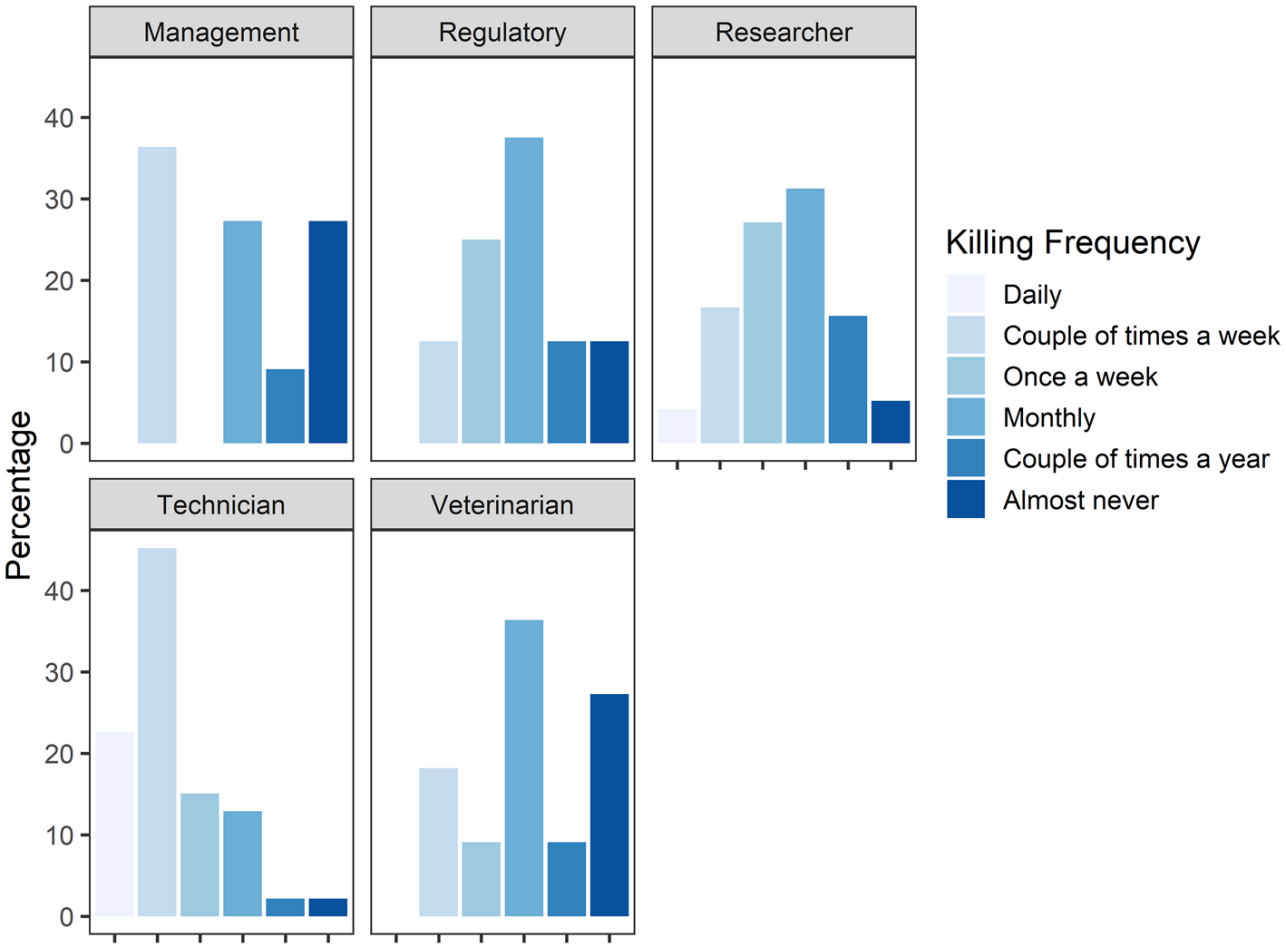
Percentage distribution (%) of reported Schedule 1 killing frequency for respondents within each defined job role. The number of respondents varied for each job role: management n = 11; regulatory n = 8; researcher n = 96, technician n = 93, veterinarian n = 11.

Only 10.0% of respondents stated that their research institute provided access to all listed Schedule 1 (ASPA) killing methods for killing non-foetal laboratory rodents. Participants reported that the Schedule 1 killing of mice predominately occurred a couple of times a week (30%) whereas for rats it was less common, reported as once a week (33.3%). Overall (n = 219), blunt force trauma was the least available method (12.3%), whilst cervical dislocation was the most widely available (88.1%). Exposure to CO_2_ (78.5%) and overdose with anaesthetic (70.8%) were available to most respondents. In the case of exposure to CO_2,_ respondents who predominately worked with mice had the greatest access to this method (81.1%, n = 154/190), compared to those working with rats (62.5%, n = 15/24). A similar pattern was observed for cervical dislocation, with greater availability for those working with mice (93.2%, n = 177/190) compared to rats (54.2%, n = 13/24). Due to the low number of participants working with hamsters and gerbils (1 respondent each) and participants working with other species (3 respondents; 1 including pigs and 2 reporting a mixture of all species) characterisation of method availability was not possible. Cervical dislocation was available for 100% of those working in a managerial and regulatory role (Table 1), and in fact, all methods bar blunt force trauma were available to individuals in a regulatory role (Table 1). Interestingly, overdose of anaesthetic was available to 100% of veterinarians and regulatory role individuals, but only 75% of researchers and 59% of technicians, despite both having primary responsibility in the killing of laboratory rodents compared to other job roles, as illustrated in Fig. 2.

**Table 1:** Availability of Schedule 1 methods and use according to job role.

Method	Available	Management	Regulatory	Researcher	Technician	Veterinarian
Cervical dislocation	Yes	11	8	82	82	10
	Total	11	8	96	93	11
	% Yes	100.0	100.0	85.0	88.0	91.0
CO_2_	Yes	9	8	83	65	7
	Total	11	8	96	93	11
	% Yes	81.8	100.0	86.0	70.0	64.0
Overdose of anaesthetic	Yes	9	8	72	55	11
	Total	11	8	96	93	11
	% Yes	81.8	100.0	75.0	59.0	100.0
Blunt force trauma	Yes	1	1	8	12	5
	Total	11	8	96	93	11
	% Yes	9.1	13.0	8.3	13.0	45.0

A majority (60.7%, n = 133/219) of respondents reported conducting Schedule 1 killing of laboratory rodents either once or a couple of times a week or daily, with the remainder reporting either monthly, a couple of times a year or almost never. Technicians appeared to have primary responsibility in the killing of laboratory rodents compared to other job roles listed, with 82.8% (n = 77/93) of them conducting Schedule 1 killing either daily, once or a couple of times a week. Researchers were the next most likely to be responsible with 47.9% (n = 46/96) of them Schedule 1 killing a laboratory rodent either daily, once or a couple of times a week. For management (36.4%, n = 4/11), regulatory (37.5%, n = 3/8) and veterinarian (27.3%, n = 3/11) job roles, less than 40% were shown to undertake Schedule 1 killing of a laboratory rodent either daily, once or a couple of times a week. Additionally, only researchers (4.2%, n = 4/96) and technicians (22.6%, n = 21/93) reported conducting Schedule 1 killing daily (Fig. 2), with more daily activity reported in mice (12.6%) compared to rats (4.2%) (Table 2).

**Table 2:** Number of participants as a percentage (%) of respondents working with each species and the frequency of Schedule 1 killing use.

Kill frequency	Mice	Rats	Hamsters	Gerbils	Other
Daily	12.6 (24)	4.2 (1)	0	0	0
Couple of times a week	30.0 (57)	25.0 (6)	0	0	66.7 (2)
Once a week	17.9 (34)	33.3 (8)	0	100 (1)	0
Monthly	25.3 (48)	16.7 (4)	0	0	0
Couple of times a year	8.4 (16)	8.3 (2)	100 (1)	0	33.3 (1)
Almost never	5.8 (11)	12.5 (3)	0	0	0
Total	190	24	1	1	3

Numbers in brackets represent the number of respondents reporting Schedule 1 killing frequency according to species.

Of those that reported ‘exposure to CO_2_’ as an available method (78.5%, n = 172), participants were asked to provide information regarding the equipment available and procedural factors such as fill method and flow rate. We found that automated equipment was the most widely available (65.7%, n = 113/172), however more than a third of participants (41.9%, n = 72/172) reported having access to non-automated equipment. Very few reported having access to both types of equipment (14.0%, n = 24/172). Despite reporting the method as available to use, 7.0% (n = 12/172) of participants reported they were unsure of the equipment type. In terms of fill method available (i.e., top or bottom fill), 50.0% (n = 86/172) reported CO_2_ was introduced to the bottom of the chamber, 27.9% (n = 48/172) indicated that CO_2_ was introduced at the top and only 1.2% (n = 2/172) stated both types of fill method were available. Interestingly, a quarter of participants (22.7%, n = 39/172) that reported CO_2_ as available to use, selected ‘don’t know’ for the fill method. Furthermore, this was influenced by job role, where researchers were more likely not to know the fill method compared to technicians (odds ratio: 5.52 ± 2.70, Z_ratio_ = 3.49, *p* = 0.004).

**Figure 3:**
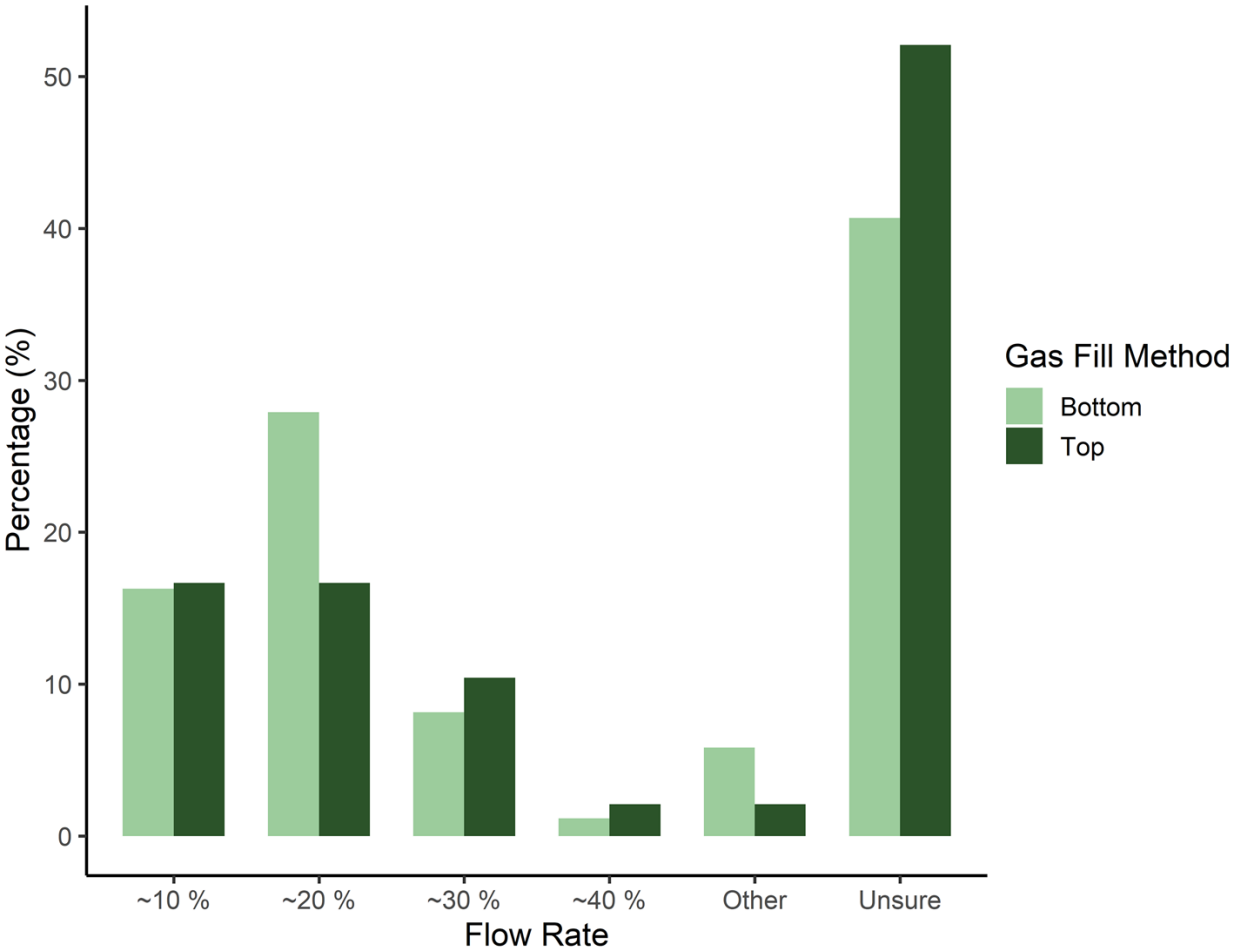
Percentage (%) of 172 participants reporting usage of different flow rates (as a percentage of the total chamber volume per minute) according to gas fill method (carbon dioxide (CO_2_) gas delivered to either the top or the bottom of the chamber) during operation of exposure to CO_2_ as a Schedule 1 killing method for adult (non-foetal) laboratory rodents.

A diverse range of flow rates for CO_2_ were reported (10–40% chamber volume per minute), but 55.2% (n = 95/172) of participants reported they were unsure of the exact flow rate used to introduce the gas into the chamber at their institution (Fig. 3). Only 18.6% (n = 32/172) of respondents reported using the recommended^4,5,18^ flow rate (i.e. 20% of the chamber volume per minute), with 14.5% (n = 25/172) of participants using a lower flow rate (< 20%) and 9.3% (n = 16/172) using higher flow rates (> 20%). However, of those using bottom fill chambers, more respondents (27.9%, n = 24/86) reported using the 20% recommended flow rate when compared to those using top fill chambers (16.7%, n = 8/48) (Fig. 3). Additionally, of those using top-fill chambers (over 50%) more respondents did not know the flow rate used (52.1%, n = 25/48), compared to 41.9% (n = 36/86) for bottom-fill users (Fig. 3). Researchers were less likely to know the flow rate compared to technicians (researcher mean likelihood: 0.00017 ± 0.34; technicians mean likelihood: 0.00003 ± 0.06: odds ratio: 0.196 ± 0.07, Z_ratio_ = 4.36, *p* = 0.001), and individuals in a managerial role (Managerial mean likelihood: 0.00000 ± 0.00 odds ratio: 11.70 ± 9.93, Z_ratio_ = 2.90, *p* = 0.03).

Most participants who had automated CO_2_ killing methods available had access to automated top-fill chambers (33.6%, n = 38/113) or bottom fill chambers (45.1%, n = 51/113). If the system was automated (bottom or top), 61.1% (n = 69/113) of participants were unsure of the flow rate, compared to 38.9% (n = 28/72) if the system was non-automated. Participants with non-automated systems were also more likely to use the recommended flow rate (26.4%, n = 19/72) compared to automated (14.2%, n = 16/113).

### Killing method preference

Overall (n = 219), irrespective of demographic factors, 57.5% (n = 126) of respondents ranked cervical dislocation as their most preferred method, while 20.5% (n = 45) preferred CO_2_, 19.6% (n = 43) chose overdose of anaesthetic and 2.3% (n = 5) preferred blunt force trauma. Score rankings in order of most preferred (1) to least preferred (4) are shown in Table 3.

**Table 3:** Mean, median, standard error (SE) of the mean and 95% confidence intervals for each schedule 1 method ranked from 1 to 4 (1 = most preferred; 4 = least preferred).

Method	Mean rank	Median rank	SE	95% CI’s
Cervical dislocation	1.60	1	0.05	[1.50, 1.71]
Overdose of anaesthetic	2.23	2	0.05	[2.13, 2.34]
Carbon dioxide	2.33	2	0.06	[2.21, 2.45]
Blunt force trauma	3.84	4	0.04	[3.76, 3.91]

**Figure 4:**
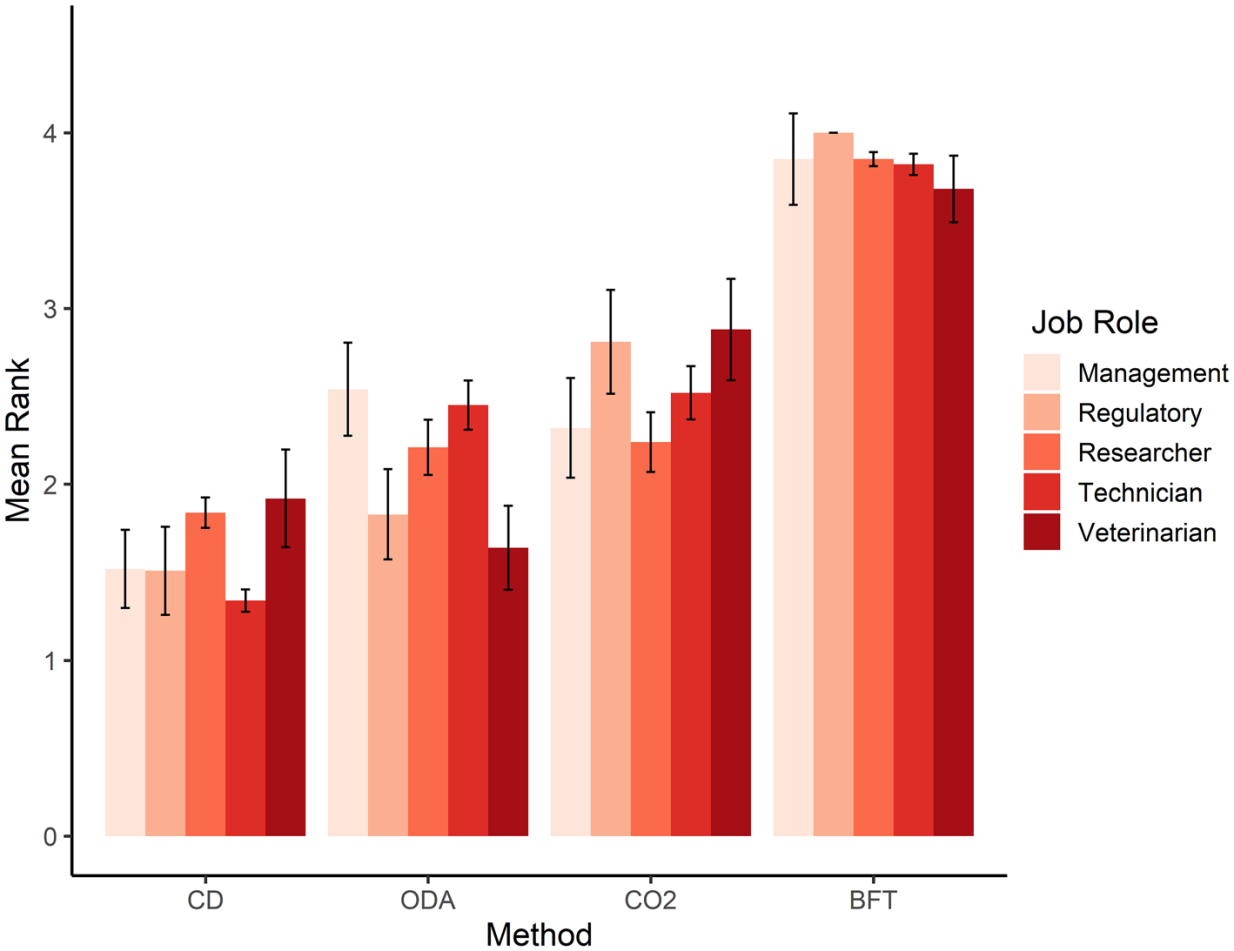
Mean ranking order (between 1 and 4 with 1 being most preferred and 4 being least preferred) of individual preference for each schedule 1 method according to current job role, BFT = blunt force trauma, CD = Cervical dislocation, CO_2_ = exposure to carbon dioxide, ODA = overdose of anaesthetic.

We found no effect of current role or job sector for ranking of exposure to CO_2,_ blunt force trauma or cervical dislocation (Fig. 4), however veterinarians were more likely to rank overdose of anaesthetic higher in terms of preference than personnel in a management (Z_ratio_: 2.85, *p* = 0.036) or technical position (Z_ratio_ = 3.531, *p* < 0.01) (Fig. 4).

**Figure 5:**
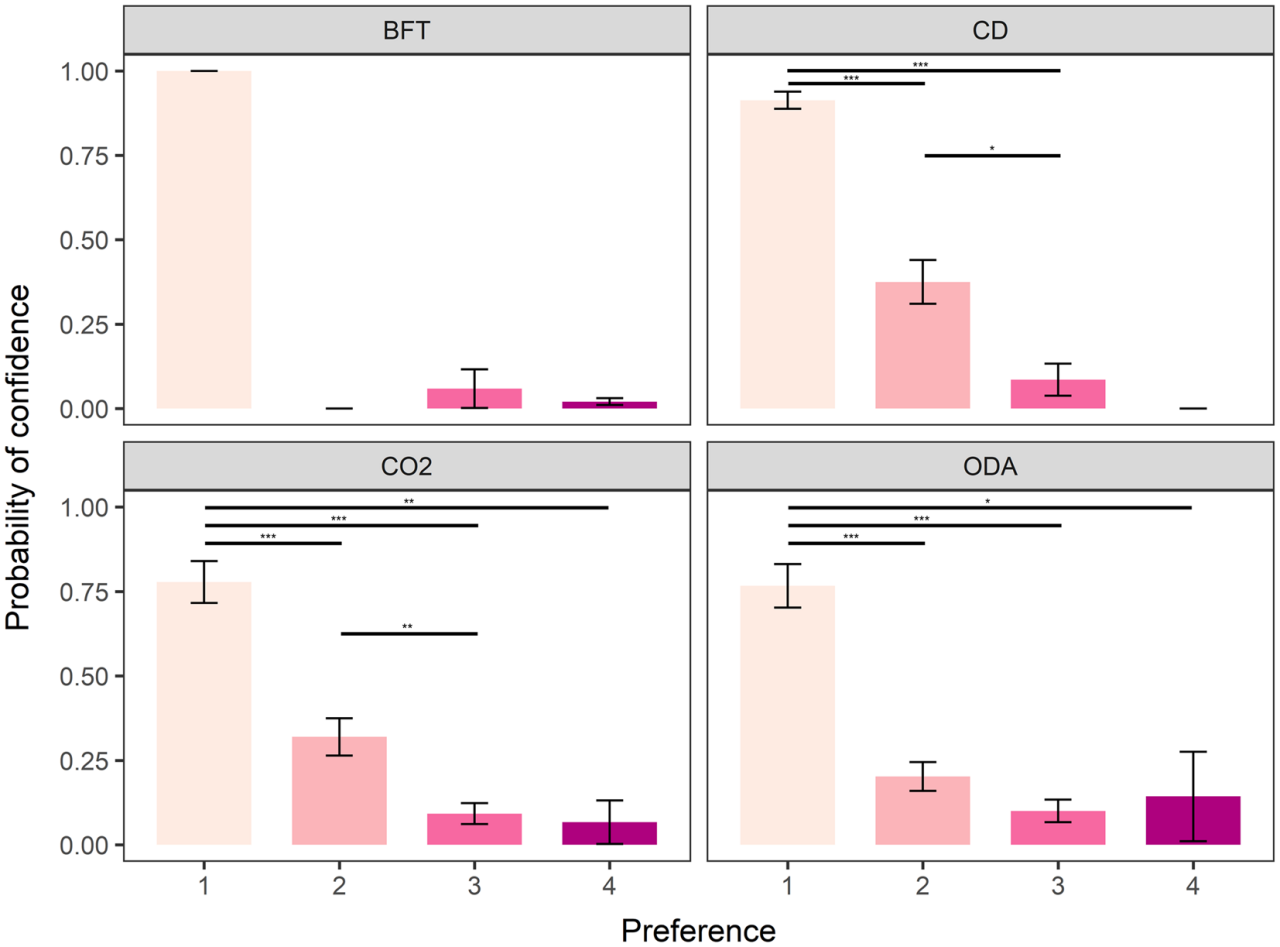
Mean (± SE) probability of participants reporting confidence according to preference ranking order (between 1 and 4 with 1 being most preferred and 4 being least preferred) for each Schedule 1 method. BFT = blunt force trauma, CD = Cervical dislocation, CO_2_ = exposure to carbon dioxide, ODA = overdose of anaesthetic. *p < 0.05, **p < 0.01, ***p < 0.001.

Furthermore, we found an effect of preferred method on the likelihood of participants reporting confidence in Schedule 1 killing methods. Participants were more likely to report high confidence for a given method if it was the most preferred (rank 1) method (blunt force trauma (Χ^2^(3) = 34.649, *p* < 0.001), carbon dioxide (Χ^2^ (3) = 71.07, *p* < 0.001), cervical dislocation (Χ^2^ (3) = 118.28, *p* < 0.001) and overdose of anaesthetic (Χ^2^ (3) = 63.16, *p* < 0.001)). However, we found that participants were significantly less likely to report strong confidence in methods ranked second through to fourth for cervical dislocation, CO_2_ and overdose of anaesthetic (Fig. 5). However, we found no effect of role, sector, or frequency of killing on the likelihood of reporting confidence in any Schedule 1 methods.

We also explored whether method availability affected confidence. This was important given that only 10% of respondents stated that all Schedule 1 methods were available for use at their institution and only 2.3% of respondents stated confidence in all four methods. We found an effect of method availability on confidence of respondents across all four methods (blunt force trauma (odds ratio: 0.083 ± 0.066, Z_ratio_ = − 3.145, *p* = 0.0017), CO_2_ (odds ratio: 0.198 ± 0.104, Z_ratio_ = − 3.077, *p* = 0.0021), cervical dislocation (odds ratio: 0.116 ± 0.067, Z_ratio_ = − 3.752, *p* = 0.0002) and overdose of anaesthetic (odds ratio: 0.286 ± 0.131, Z_ratio_ = − 2.725, *p* = 0.0021)). These findings demonstrate that respondents were more likely to report confidence for available methods. In addition, when accounting for method availability, we found a greater probability of confidence in cervical dislocation across technicians compared to researchers (odds ratio: 0.261 ± 0.106, Z_ratio_ = − 3.296, *p* = 0.009), but no difference between other roles from pairwise comparisons.

### Killing method properties according to method

Respondents were also asked to rank eleven properties in order of importance for their preferred killing method. The predominant characteristic influencing preference for a particular killing method was humaneness, and 63.9% (n = 140) of all respondents (n = 219) ranked this as the primary factor. However, other selected properties included fast application (16.9%, n = 37), reliability (8.2%, n = 18), easy to perform (5%, n = 11), ‘operator perception’ (1.4%, n = 3), high-throughput (1.4%, n = 3), minimal contact (0.9%, n = 2), no equipment required (0.9%, n = 2), safe for operator (0.9%, n = 2) and cost effective (0.5%, n = 1).

**Figure 6:**
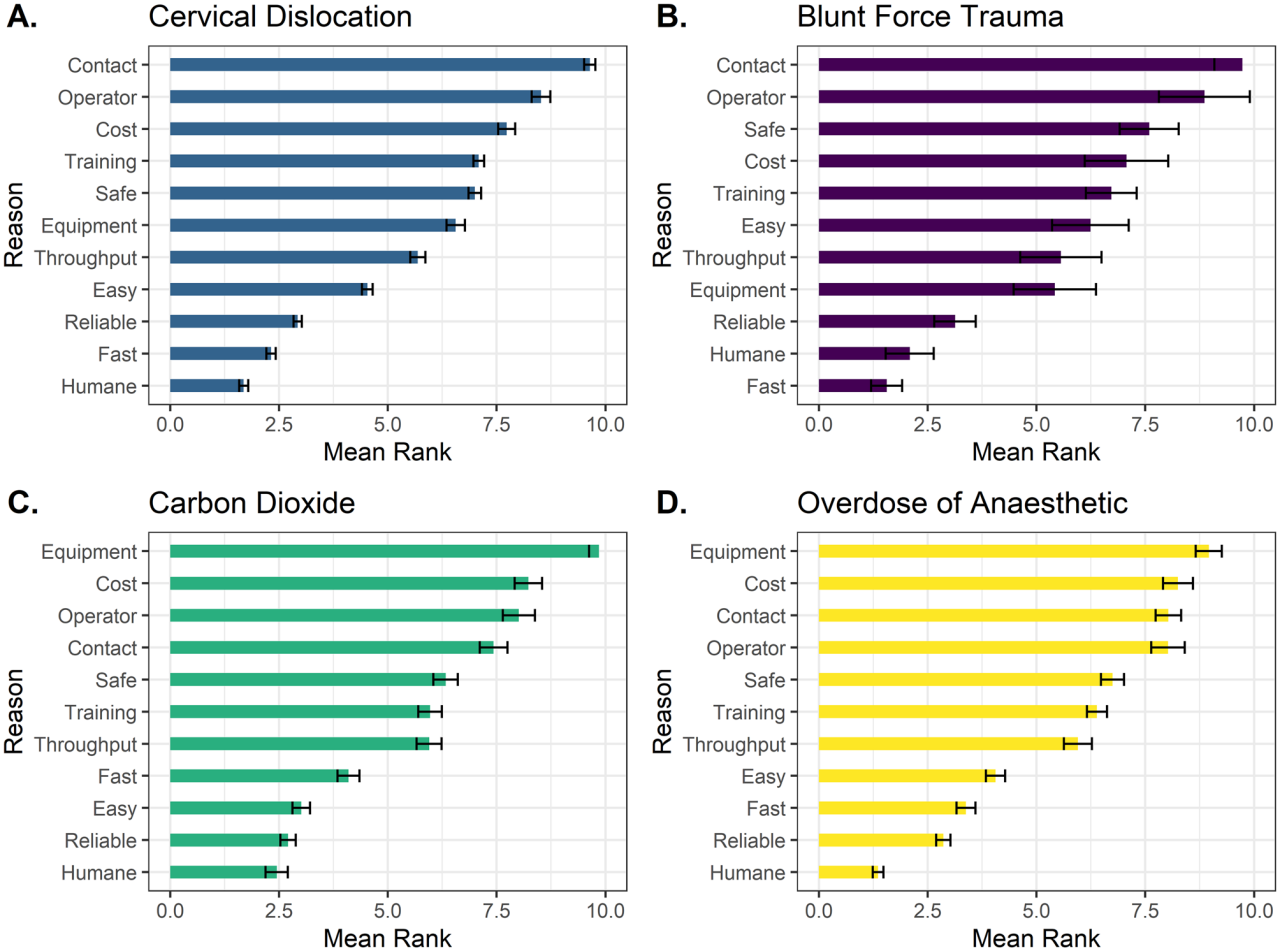
Mean (± SE) rank for each reason underpinning preferred Schedule 1 methods. Reasons were ranked in order of importance from 1 to 11 with 1 being most important and 11 being least important (219 respondents). Please note y axis of characteristics vary for each method, allowing for them to listed in order of importance for each method.

We determined whether the mean rank attributed to each property was affected by the preferred killing method of participants. Individuals who selected CO_2_ as their preferred method ranked humaneness as less important compared to individuals who preferred overdose of anaesthetic (Z_ratio_ = 3.91, *p* < 0.001) and cervical dislocation (Z_ratio_ = 2.82, *p* = 0.02). Therefore, overdose of anaesthetic was ranked as the most humane method (Fig. 6). We also found a significant difference in the ranking of fast application. Respondents preferring physical methods such as blunt force trauma and cervical dislocation were more likely to rank fast application as important compared to overdose of anaesthetic (Z_ratio_ = 4.38, *p* < 0.001 and Z_ratio_ = 4.40, *p* < 0.001 respectively), and exposure to CO_2_ (z ratio = 5.82, *p* < 0.001 and Z_ratio_ = 6.51, *p* < 0.001 respectively). Indeed, for blunt force trauma, fast application was ranked as the most important factor, rather than humaneness which was selected for all other methods (Fig. 6). When considering ease of application, individuals whose preferred method was CO_2_ exposure valued easy application more than those who preferred blunt force trauma (Z_ratio_ = 3.96, *p* < 0.001), cervical dislocation (Z_ratio_ = 6.45, *p* < 0.001) and overdose of anaesthetic (z ratio = 3.49, *p* < 0.01). For physical methods i.e., cervical dislocation and blunt force trauma, participants ranked ‘no equipment required’ as more important compared to exposure to CO_2_ (Z_ratio_ = 10.65, *p* < 0.001, Z_ratio_ = 4.55, *p* < 0.001 respectively) and overdose of anaesthetic (Z_ratio_ = 6.55, *p* < 0.001, z ratio = 3.57, *p* < 0.001 respectively). Minimal training required was ranked as a more important factor for individuals with a preference for exposure to CO_2_ and overdose of anaesthetic compared to cervical dislocation (Z_ratio_ = 3.75, *p* < 0.001, Z_ratio_ = 2.73, *p* = 0.033 respectively). Finally, respondents whose preferred method was CO_2_, were more likely to rank non-contact as more important compared to individuals who preferred blunt force trauma (Z_ratio_ = 3.21, *p* < 0.01) and cervical dislocation (Z_ratio_ = 6.51, *p* < 0.001). For all other properties we found no differences in the mean ranking order across the four Schedule 1 methodologies (Fig. 6). Factors such as cost, operator perception and safety were all ranked relatively low in terms of importance across all methods, in contrast to method reliability which was identified as important across all methods. Interestingly, throughput ranked intermediary in terms of importance across all methods, despite CO_2_ being the only high-throughput method (Fig. 6).

## Discussion

These findings provide new information regarding the availability and use of Schedule 1 methods permitted for killing laboratory rodents across UK-based research institutions and provides valuable insight into the perspectives of personnel utilising them—topics in which few data are currently available. Two previous studies have obtained responses from laboratory personnel regarding killing practices. However, one was focused on the consequences of killing on human quality of life rather than animal welfare consequences^19^, and the other solely focused on attitudes towards CO_2_ killing with no focus on procedural aspects or other permitted methods^20^. Despite four methods being permitted for use in Schedule 1 ASPA legislation, we found that only 10% of respondents had all four methods available for use for killing adult laboratory rodents. While any individual’s preference for method selection is multifaceted, we found that individuals report greater confidence in a method if it is their preferred option, with a reduced likelihood of reporting confidence for approaches that they ranked lower. However, it is unlikely that an individual’s confidence and preference for a method are independent from one another, and instead their relationship is likely intertwined and bidirectional. Nonetheless, method unavailability presents a major concern for operator safety and animal welfare; if relocated to a different research institution, an individual may be forced to utilise a method they lack confidence in which has the potential to lead to poor animal welfare outcomes if performed incorrectly. After all, as confidence in a method decreases, the risk of incorrect application is likely to increase. This is further exacerbated by our findings that method availability was influenced by job role. We found that overdose of anaesthetic was available to all veterinarians and individuals holding a regulatory role, but despite technicians and researchers having primary responsibility for killing, it was unavailable to 41% of technicians and 25% researchers. Overdose of anaesthetic in the veterinary profession is considered the gold standard, whereby companion animals are typically administered a barbiturate anaesthetic agent intravenously^21^ and therefore it is perhaps surprising that this method was reported to be less available to those holding primary responsibility for killing laboratory rodents. However, whether overdose of anaesthetic offers the most humane option for killing laboratory rodents is debated^22,23^. The most common agent used for overdose of anaesthesia for laboratory rodents is injectable pentobarbital^23,24^. Unlike companion animals, the placement of intravenous catheters is extremely difficult in laboratory rodents and therefore this approach involves intraperitoneal injection of barbiturate agents following restraint of the animal^14^. This has obvious welfare consequences including the stress associated with restraint, pain and discomfort upon introduction of the agent into the peritoneal cavity and increased risk of perforating the bowel^23,25,26^. Nonetheless, the inclusion of all four methods in legislation suggests that they should all be available for use across institutions and access should not be dependent upon factors such as job role.

Our results also highlight a lack of knowledge and consistency surrounding the procedural aspects of exposure to carbon dioxide (CO_2_) gas. A quarter of participants didn’t know the fill method of the gas chamber (i.e. whether the gas was introduced to the bottom or the top) and more than half didn’t know the exact flow rate of gas delivery to the chamber (this issue was more prevalent amongst researchers compared to technicians or those in a managerial role). This is extremely concerning given that the welfare consequences and potential to experience negative sensations such as dyspnoea and pain is highly dependent on the concentration of CO_2_ inside the chamber prior to the animal losing consciousness^9,27,28^. CO_2_ is heavier than air and therefore the fill method is crucial in determining dissipation and gas concentration inside the chamber^5,27^. Of respondents that knew the fill method, most users employed bottom filling CO_2_ equipment. This is surprising, given that top filled chambers are considered best practice^5^ since introducing the gas to the top of the chamber prevents the accumulation at the bottom of the chamber and potential exposure of conscious rodents to nociceptive concentrations. Although top fill is considered best practice^5^, this is not outlined in existing legislation. Instead, Schedule 1 states ‘exposure to carbon dioxide gas in a rising concentration’ which perhaps creates ambiguity surrounding the appropriate fill method. It is possible that the inclusion of the word ‘rising’ suggests that CO_2_ should be introduced at the bottom of the chamber. Furthermore, it is widely thought that the recommended flow rate should fall between 10 and 30% (recommended is 20%)^4,5^ of the chamber volume per minute in order to avoid concentrations capable of eliciting pain (above 40%) before loss of consciousness^9^. However, only 18.6% of respondents reported using the recommended^4,5^ flow rate (i.e., 20%), with 14.5% of participants using a lower flow rate (< 20%) and 9.3% using higher flow rates (> 20%). We also found a lack of knowledge among participants regarding automated versus non-automated systems. Participants using non-automated systems were more likely to apply the recommended flow rate (26.4%) compared to automated (14.2%) users. Therefore, we suggest that with automation comes a lack of training and knowledge, which becomes problematic if failure to adopt the correct flow rate occurs in response to a failure in automation. Collectively, these findings highlight significant operational gaps in knowledge surrounding exposure to CO_2_ in rodent Schedule 1 killing which increase the risk of negative welfare consequences, highlighting that current training programmes are inadequate. Effective training could take the form of independent quality-controlled training programmes to ensure consistency in method application and animal welfare standards during killing across research institutions.

Overall, we found that the most widely preferred killing method for rodents was cervical dislocation and not CO_2_ as reported by a recent survey^20^. This method involves the separation of the cervical vertebrae resulting in lethal trauma to the spinal cord and is commonly performed by placing the finger (manual) or an instrument (mechanical) behind the base of the skull whilst pulling the tail firmly to achieve rapid separation of the high cervical vertebrae. This technique requires speed and accuracy (regarding dislocation location) to ensure method success^14,15,29^. Therefore, operator confidence is crucial for ensuring rapid separation of the cervical and not thoracic region of the spinal cord. As with all other methods, operator confidence was highly dependent upon method availability, whereby participants were more likely to report confidence in a method if it was available for use at their institution likely due to more opportunity to practice. However, when accounting for method availability we also found that confidence in performing cervical dislocation was dependent upon job role, with technicians reporting a higher likelihood of confidence in cervical dislocation than researchers. This is concerning given that researchers, after technicians, have frequent responsibility for killing laboratory rodents, and suggests a further need for dedicated training amongst the research community.

We were interested in determining the reasons behind individuals’ preferences for method selection and therefore asked participants to rank the importance of eleven factors. Most participants selected humaneness as their primary reason for method selection, however other factors such as fast application, reliability and ease were also ranked highly. For physical methods (cervical dislocation and blunt force trauma) a lack of dedicated equipment played a role in method selection. By contrast, for users of CO_2_, reasons for preference included its ability to provide a non-contact approach, ease of application and its requirement for minimal training. These attitudes towards this technique may be misplaced; although CO_2_ application can be considered ‘easy’ in terms of placing an animal in a chamber and switching on the gas supply, it remains vital that users know the correct flow rates, fill methods and safe operation of the equipment to ensure method success and minimise unnecessary suffering. Interestingly, although CO_2_ is the only permitted method capable of high throughput, this was not reflected in our survey i.e., high throughput was not ranked as a higher factor for CO_2_ compared to other methods. This suggests that its high throughput advantage may be underappreciated which provides impetus for further scrutiny of its approved status in Schedule 1 legislation. Significant welfare concerns surrounding its use remain^4–6^, however a high-throughput alternative with better welfare outcomes is yet to be developed. It is possible that individuals have been discouraged from employing CO_2_ irrespective of its high-throughput advantage given the significant negative attention it has received over recent years. In a recent survey, 51% of participants felt there were alternative methods considered an improvement over CO_2,_ however this was not reflected in individual attitudes, which remained ambivalent overall^20^. Therefore, it remains unclear whether the advent of a suitable alternative would influence personal preferences and perhaps promoting a move away from physical methods. The method actually used and their effects on those applying them is important given that burnout and compassion fatigue have been found to be most prevalent in individuals employing physical killing methods^19^ and availability of a reliable and humane non-contact method could improve the wellbeing of laboratory animal personnel.

A key finding is that method and equipment availability across institutions is not fixed and there are worrying gaps in knowledge surrounding correct application of carbon dioxide killing. The current survey collected anonymous responses and therefore we were unable to determine the representative sample of the wider population. Therefore subsequent work should focus on obtaining responses across the entire sector and utilise open text qualitative responses to further explore operator motivations behind method choice. Future efforts to improve the welfare of laboratory rodents at killing should focus on the development and optimisation of standard operating procedures and quality-controlled training programmes to promote best practice and ensure consistency. Further, there is a need for dissemination of information and training amongst the research community to increase confidence and knowledge supporting choice of killing method. We emphasise the importance of method availability and advocate for continuous review of existing protocols and guidance to ensure best practice is reflected in legislation. We also advocate for careful review of terminology, alongside ongoing scientific efforts to develop novel approaches to killing that prioritise animal welfare while meeting the needs of the scientific community.

## Methods

### Participants

The target population of this survey was individuals that work for a research institution in the United Kingdom and who predominantly work with laboratory rodents (mice and/or rats). The survey was piloted internally with target respondents (e.g. technicians) to ensure clarity in question phrasing and interpretation prior to dissemination. Participants were invited to take part via dissemination of a link across various animal care and welfare and 3Rs organisations (NC3Rs, IAT, LASA, LAVA). All participants that completed the survey were included in the analysis and the research was approved by the University of Glasgow MVLS College Ethics committee (reference: 200200180). All experiments were performed in accordance with relevant guidelines and regulations.

### Online survey

The online survey was hosted through Qualtrics^30^ and was open between August 2021 and January 2022 (for full survey see Supplementary Materials). The survey was fully anonymous, and informed consent was obtained prior to commencement to the online questions. The first part of the survey obtained basic demographic information from participants across four question including which sector they currently worked in, their current role, their number of years’ experience working with laboratory rodents and the species they predominantly work with. The second part consisted of identifying respondents’ preferences for the permitted methods outlined in Schedule 1 of ASPA^2^. These included how often they euthanise laboratory rodents and which methods are available at their host institution. If applicable, the questionnaire asked what type of CO_2_ system was available, what protocol is employed for filling the induction chamber with CO_2_ and at what flow rate (as a percentage per minute), and how important respondents thought it was to protect welfare, that the animal is monitored during CO_2_ exposure and how important they think it is that the chamber is transparent. Respondents were then asked to rank the four legally available Schedule 1 methods according to their personal preference for killing an individual rodent, as well as ranking the methods based on their confidence in performing them successfully. Similarly, we asked for a ranking of the four methods according to their preference for killing multiple rodents (e.g., groups of 10 +) simultaneously or consecutively. Finally, respondents ranked several potential and pre-defined decision factors in order of importance for informing their preferences.

### Data analysis

Data was exported from Qualtrics as an Excel file format. All statistical analyses were conducted in R^31^ and R Studio (version 1.3.1093^32^). Only responses from individuals who consented to and completed the full survey were included in the analysis. Responses were fully anonymous and therefore in accordance with the General Data Protection Regulation^33^. All data was collated and processed within R using the tidyverse package^34^. All graphical summaries were created using ggplot2^35^. Ranked data was analysed using Cumulative Link Models (CLMs) using package ordinal^36^ and RVAideMemoire^37^ to compare mean ranks with the threshold set to equidistant. Exploration of the influence of demographic factors was performed via models including fixed factors such as job role (5 levels), job sector (4 levels), experience (4 levels) and frequency of performing Schedule 1 killing (6 levels). Statistical significance was based on *p* < 0.05 threshold on the Χ_2_ statistical test. Pairwise comparisons were reported using estimated marginal means via the emmeans package, with P values adjusted for multiplicity using the Tukey method^38^ where non-significant results are not reported.

## Supplementary Information

Supplementary Information.

## Data Availability

All data generated in and analysed during the current study will be publicly available in the University of Glasgow’s research online data repository (http://dx.doi.org/10.5525/gla.researchdata.1305).
