# Supplementary material for: The perspectives of UK personnel towards current killing practices for laboratory rodents
Source: Sci Rep. 2023 Mar 23;13:4808. doi: 10.1038/s41598-023-31808-0 (PMC10036518; doi:10.1038/s41598-023-31808-0)
Supplement: Supplementary file 1 — Supplementary Information. [file 41598_2023_31808_MOESM1_ESM.pdf]

Supplementary Materials for;

“The perspectives of UK personnel towards current killing practices for laboratory rodents”

University of Glasgow | College of Medical, Veterinary & Life Sciences

Protocols for laboratory rodent euthanasia

The aim of this study is to investigate the existing protocols and preferences research institutions use across the UK to euthanise laboratory rodents. We invite you to complete a short questionnaire. The research is being conducted as part of a wider project investigating potential alternative, humane methodologies for rodent euthanasia. Taking part is entirely voluntary and the questionnaire will take around 5 minutes to complete. To participate you must be over 18 and work for an established research institution (e.g., academia or industry) and have experience working with laboratory rodents.

Completion of the survey is anonymous, and we will not collect any identifiable information from you for more information about data processing see (hyperlink to privacy notice). Nevertheless, all study data will be held in accordance with The General Data Protection Regulation (2018). The data will be stored in archiving facilities in line with the University of Glasgow retention policy of up to 10 years.
https://www.gla.ac.uk/myglasgow/dpfoioffice/a-ztopics/dataretentionforresearchdata/

This study has been approved by the University of Glasgow College of Medical Veterinary and Life Sciences Ethics Committee in collaboration with Dr Matt Leach, Newcastle University.

Contact for further information:
Dr Matt Leach & Dr Jasmine Clarkson – mvls-schedule1survey@glasgow.ac.uk

Consent

I confirm consent to the following:

- I confirm that I have read and understood the information provided
- I understand that all data and information I provide will be anonymous and will be seen only by study researchers
- I confirm that I agree that data will be stored for at least 10 years in University archiving facilities in accordance with relevant Data Protection policies and regulations.
- I understand that if I withdraw from the study, my data collected up to that point may be retained and used for the remainder of the study.

I confirm that I consent to take part in the study. ☐

Part 1: Demographics

1. Which sector(s) do you currently work in?
    a. Academic
    b. Pharmaceutical
    c. Contract Research Organisation
    d. Other (please specify)

2. What best describes your current role?
    a. Technician
    b. Veterinarian
    c. Researcher
    d. Regulatory
    e. Other (please specify)

3. How many years experience do you have working with laboratory rodents?
    a. 0-1 years
    b. 2-5 years
    c. 6-11 years
    d. 12-17 years
    e. 18-23 years
    f. 24 + years

4. What laboratory species do you predominantly work with?
    a. Mice
    b. Rats
    c. Hamsters
    d. Gerbils
    e. Guinea pigs
    f. Other (please specify)

Part 2: Identification and preference of euthanasia methods used

For the following questions, please answer based on the species you primarily work with (as answered in question 5).

5. On average, how often do you euthanise laboratory rodents?
    a. Daily
    b. Couple of times a week
    c. Once a week
    d. Monthly
    e. Couple of times a year
    f. Almost Never

6. From the listed Schedule 1 (ASPA) killing methods listed for non-fetal, embryonic laboratory rodents, which are **available for use** at your institution to euthanise laboratory rodents? (please tick all that apply)
   a. Overdose of an anaesthetic
   b. Exposure to carbon dioxide gas in a rising concentration
   c. Dislocation of the neck (with sedative for rodents over 150g)
   d. Concussion of the brain by striking the cranium

7. If identified as available (as answered in question 6), what type of equipment is available for carbon dioxide euthanasia?
   a. Fully automated carbon dioxide system
   b. Non-automated carbon dioxide system
   c. Don't know
   d. Other (please specify)

8. If identified as available (as answered in question 6), what protocol is employed for filling the induction chamber with carbon dioxide at your institution?
   a. Carbon dioxide is introduced to the top of the chamber
   b. Carbon dioxide introduced at the bottom of the chamber
   c. Don't know
   d. Other (please specify)

9. If identified as available (as answered in question 6), what flow rate as a percentage of the chamber volume per minute do you introduce the carbon dioxide into the chamber at your institution?
   a. 10% - 20%
   b. 20% -30%
   c. 30-40%
   d. 40 - 50%
   e. >50%
   f. I don't know
   g. Other (please specify)

10. If identified as available (as answered in question 6), how important to protect welfare do you think it is to monitor the animals during carbon dioxide euthanasia?

☐ Not at all important ☐ Slightly important ☐ Moderately important ☐ Very important ☐ Extremely important

11. If identified as available, how important to protect welfare do you think it is that the CO_2 induction chamber is transparent?

☐ Not at all important ☐ Slightly important ☐ Moderately important ☐ Very important ☐ Extremely important

12. Based on what methods are available to you, please order the four Schedule 1 methods according to **your personal preference** for killing an **individual** rodent (1 being most preferred and 4 least preferred).
    a. Overdose of an anaesthetic
    b. Exposure to carbon dioxide gas in a rising concentration
    c. Dislocation of the neck (with sedative for rodents over 150g)
    d. Concussion of the brain by striking the cranium

13. From the listed Schedule 1 (ASPA) killing methods listed for adult laboratory rodents, which method are you most **confident** using to euthanise an **individual** rodent?
    a. Overdose of an anaesthetic
    b. Exposure to carbon dioxide gas in a rising concentration
    c. Cervical dislocation of the neck (with sedative for rodents over 150g)
    d. Concussion of the brain by striking the cranium

14. Based on what methods are available to you, please order the four Schedule 1 methods according to preference for killing **multiple (i.e. 10+)** rodents simultaneously or consecutively.
    a. Overdose of an anaesthetic
    b. Exposure to carbon dioxide gas in a rising concentration
    c. Dislocation of the neck (with sedative for rodents over 150g)
    d. Concussion of the brain by striking the cranium

15. From the reasons listed below, please rank in order of importance why the method identified as most preferred in Q12 (1 being most preferred and 11 least preferred).
    a. Humane
    b. Fast application
    c. Reliable
    d. High throughput
    e. Easy to perform
    f. Cost effective
    g. Safe for the operator
    h. No equipment required
    i. Minimal training required
    j. Minimal contact / Less hands-on
    k. Operator perception

Thank You Page

You have now completed this survey and your responses have been recorded.

If you have any further questions about this study, please do not hesitate to contact Dr Matt Leach and Dr Jasmine Clarkson via mvls-schedule1survey@glasgow.ac.uk.

Thank you, your participation in this research is greatly appreciated.
